# Development of an instrument to assess the mental health of university students: validation of the *Outcome Questionnaire-45* in a Hungarian sample

**DOI:** 10.3389/fpsyg.2024.1334615

**Published:** 2024-01-17

**Authors:** Dominika Matavovszky, Lan Anh Nguyen Luu, Orsolya Karner

**Affiliations:** ^1^Doctoral School of Psychology, ELTE Eötvös Loránd University, Budapest, Hungary; ^2^Institute of Psychology, ELTE Eötvös Loránd University, Budapest, Hungary; ^3^Institute of Intercultural Psychology and Education, ELTE Eötvös Loránd University, Budapest, Hungary

**Keywords:** *Outcome-Questionnaire-45*, cross-sectional condition assessment, university students, questionnaire, validation, OQ-39

## Abstract

The Outcome Questionnaire is a self-report questionnaire developed mainly for treatment impact assessment and monitoring of status change because it can measure the cross-sectional condition very accurately by being sensitive to small changes. The present study aimed to psychometrically evaluate and validate the instrument on a sample of Hungarian university students. 7,695 higher education students (28.6% male, 68.8% female, 1% other, *M* = 23.7, SD = 6.78) participated in the study and completed a questionnaire package (OQ-45, Beck Depression Inventory, WHO Well-being Questionnaire-5, Connor-Davidson Resilience Scale, MOS-Social Support Survey, Maslach Burnout Inventory-SS) online, developed to measure general and more specific mental health conditions. The Hungarian version of the questionnaire has a high internal consistency (Cronbach’s alpha = 0.951). Based on the confirmatory factor analysis, the original three-factor version of the instrument (due to inadequate fit indicators) did not gain support in our sample. Five subscales were identified and subjected to content analysis in the exploratory factor analysis. Our final questionnaire consists of 39 items. The full scale and the subscales show a high correlation with other questionnaires measuring similar constructs. The psychometric indicators of the questionnaire are adequate and, therefore, considered reliable. The separation of the five factors was confirmed by construct and convergent validation. The questionnaire’s psychometric properties may be worth testing in the future on a clinical sample and a sample of adults from a wider age range. The use of the measurement tool has important implications in research areas beyond therapeutic impact assessment, as it may offer a bridging solution to the methodological problems encountered in the construction of complex questionnaire packages consisting of several instruments. International findings suggest that some items in the questionnaire are particularly sensitive to cultural context, so it is crucial to use a measure adapted to the region of the study sample. Other strengths of the questionnaire include its ability to address subclinical and clinical symptoms in one dimension and provide a comprehensive cross-sectional picture of the bio-psycho-social status of individuals, which allows systematic monitoring of a large and heterogeneous population (higher education students).

## 1 The *Outcome Questionnaire-45*

Positive or negative changes in psychotherapy and psychological counseling are very difficult to quantify, and it is not easy to identify the factors that determine effective treatment. Nevertheless, there is a strong demand from professionals, clients and the health care system to monitor the impact of a given intervention and to measure the effectiveness of therapies (Goss and Rose, 2002). For this reason, impact evaluations are becoming increasingly unavoidable and, in parallel with the spread of psychotherapies, they are also an important part of psychological research (Papp and Péley, 2015).

Measuring the effectiveness of therapies has a long history, dating back to the 1950s. Eysenck (1952), one of the first researchers on the subject, expressed a very skeptical view of positive effects. This was repeatedly refuted in later studies in the 1970s using a more sophisticated methodology (Bergin, 1971). With the spread of cognitive and behavioral trends, more and more factors (e.g., therapist role, client characteristics, etc.) were included in impact studies (Lambert, 2013). Although the research methodology was very heterogeneous, the effectiveness of the therapies was clearly demonstrated in about two-thirds of the cases. At the same time, however, 10–15% of clients experienced deterioration as a result of the interventions (Lambert and Ogles, 2004), creating a need for professionals to focus on preventing and reducing potential negative outcomes in addition to achieving positive change (Lambert et al., 2005). A self-report questionnaire, designed to consistently monitor the therapeutic process, can be used to eliminate potential biases in the assessment of treatment effectiveness due to therapist bias.

The *Outcome Questionnaire-45* (OQ-45; Lambert et al., 1996) primarily supports the work of therapists and helping professionals by providing a systematic picture of the client’s functioning and by allowing the measurement of therapeutic effectiveness by monitoring the client’s condition and assessing changes in the process (by identifying improvement and deterioration). Accordingly, the instrument can be used repeatedly at the beginning, during, and at the end of treatment.

The questionnaire consists of 45 items (9 of which are reversed) all of which cover the spectrum of mental disorders, symptoms, and stress disorders. The respondent is asked to rate each item on a 5-point Likert scale (1: never, 5: almost always) based on their experience over the past week. The total score calculated from the responses gives an indication of the client’s overall health, and the subscale scores give an indication of functioning in different areas. Of the 45 items, 4 items have a signaling function (of which item 8 indicates suicidal tendencies, items 11 and 32 indicate substance abuse, and item 44 indicates aggression). The strengths of the measure are that it is able to discriminate (but not diagnose) clinical from subclinical symptoms, it is inexpensive, easy and quick to administer, and sensitive to small changes (Lambert and Ogles, 2004). As it uses a common measure of mental health, the results obtained may also be suitable for describing cross-sectional conditions.

These advantages of the questionnaire can be further exploited in the case of higher education students, as the mental health of this large and heterogeneous target group can be measured easily and quickly. A comprehensive study of this population is of particular importance and relevance in several respects. The high prevalence of anxiety and depressive symptoms among young people (e.g., Kessler and Wang, 2008; Gallagher, 2016) the life management problems and chronic diseases that develop during this period clearly indicate an increasingly vulnerable period both physically and psychologically (e.g.: Benczúr et al., 2014; Zhang et al., 2019). This may be due, among other things, to normative crises caused by the life tasks to be tackled in young adulthood, e.g.: formation of intimate relationships, separation from parents (Erikson, 1959). Social changes in recent decades (e.g., expanded and unpredictable opportunities for learning, work and mobility) present new mental challenges to which adaptation requires additional mental energy (Arnett, 2004). In addition, those involved in higher education are also exposed to external and internal sources of stress associated with studying (e.g., academic performance, financial resources, student work (e.g., Bewick et al., 2010). Overall, therefore, there are a number of risk factors for mental health that warrant screening and early detection of mental and physical illness. This calls for systematic studies that can measure both the developmental and psychological characteristics of emerging adulthood (e.g., identity exploration, feeling in between) and coping responses to stressors created by the university environment. Effective and timely intervention not only reduces the burden on the university counselor and the healthcare system, but can also have a positive impact at a socio-economic level (Urbán, 2017).

The questionnaire has the following three subscales: (1) *Symptom Distress* (SD) (2) *Interpersonal Relationships* (IR) (3) *Social Roles* (SR). Lambert argues for the existence of these factors and their distinction from each other even before the development of the OQ-45, referring to Strupp and Hadley’s (1977) three-factor theoretical model of mental health (Lambert, 1983).

The *Symptom Distress* factor includes various aspects of intrapsychic functioning. Its items measure symptoms of depression and anxiety, the levels of subjective distress, emotion management and emotion regulation, other somatic complaints and concentration problems. The *Interpersonal Relationships* dimension examines relationships with others, family conflicts, and feelings of loneliness. The *Social Roles* subscale explores difficulties, identification problems, or role conflicts related to different social roles, e.g., employee, student. For each of the three factors, a high score indicates a problem in that given area, while a low score per subscale indicates less subjective distress, harmony in interpersonal relationships or appropriate role management.

The instrument has been widely used in several countries, and translated into more than 20 languages, with psychometric analyses conducted in Germany, the Netherlands, Italy, Sweden, China and other countries (Amble et al., 2014). Replicated US studies have demonstrated the high internal consistency of the questionnaire (Lambert and Ogles, 2004), with the highest reliability values for the total score and the Symptom Distress subscales in both academic (OQ-45_total_ = 0.93; SD = 0.92; IR = 0.74; SR = 0.7) and clinical (OQ-45_total_ = 0.93; SD = 0.91; IR = 0.74; SR = 0.71) samples. For the full scale, as in the original, the translated versions have good reliability (Cronbach’s alpha = 0.91–0.96) and test–retest indices (Pearson’s *r* = 0.84–0.91) and show strong correlations with questionnaires measuring similar constructs (e.g., Lambert et al., 1996; Umphress et al., 1997). However, other psychometric properties of the instrument vary considerably across countries, including variability in mean scores in clinical (78.7–83.1) and non-clinical (38.7–61) samples, cut-offs for indicating treatment change (Amble et al., 2014), and variability in the number of factor structures. Subsequent studies in the US (e.g., Mueller et al., 1998; Chapman, 2003; Bludworth et al., 2010) and in other countries (e.g., Lo Coco et al., 2008; Tabet et al., 2020) have not confirmed the existence of the previously hypothesized three dimensions. The non-reproducibility of the original structure has led researchers to construct alternative models (e.g., de Jong et al., 2007; Rice et al., 2014; Simon et al., 2015). Exploratory analyses have shown considerable variation in the structure of the factors, with four (Lo Coco et al., 2008) five (de Jong et al., 2007), nine (Chapman, 2003) and ten (Wennberg et al., 2010) factor solutions were obtained (cited in Amble et al., 2014). Beyond these, we also find single-level (e.g., de Jong et al., 2007) and multi-level structures (e.g., Bludworth et al., 2010; Tabet et al., 2020). The variation in the results of international research findings draw attention to the differences and cultural embeddedness of health services. A sample of university students is often used for international validity testing of the OQ-45 (e.g., Beretvas et al., 2003; Chapman, 2003) partly because the questionnaire, originally designed to measure treatment effect, is able to accurately detect change at the subclinical level. This means that it can be used as a measure in university counseling centers [where well-functioning individuals are also cared for Erickson Cornish et al. (2000)] and the heterogeneous subclinical university population can be considered a good control group compared to the clinical sample.

The aim of our study was to validate the Outcome Questionnaire (OQ-45; Lambert et al., 1996) in a Hungarian sample, contributing to the validation work done in other countries. The questionnaire could have a wide range of applications in Hungary. On the one hand, we hope that the use of this change-sensitive measure will facilitate the work of psychological centers, healthcare institutions with limited financial and human resources, and university counselors in terms of screening and intervention. In addition, we feel it is important to emphasize that, in addition to its practical use, the validated questionnaire can also play a niche role in research. The OQ-45 instrument provides a comprehensive cross-sectional picture of the individual’s bio-psycho-social status. The main goal of our research group is to conduct systematic large-scale annual surveys among Hungarian university students in order to better understand the relationship between the factors determining the mental state of the population (e.g.: developmental and psychological processes, social changes, characteristics of social relationships) and to react to changes and results that occur in the meantime. To do this, however, we need a large sample. Because of the shortness of the OQ-45 questionnaire, participants are more likely to be motivated to complete it, thus limiting the difficulties associated with drop-out. The Hungarian adaptation of the questionnaire therefore aims to meet both these practical and scientific needs.

Although the high reliability of the original questionnaire has been confirmed by several international studies (e.g., Qin and Hu, 2008; Wennberg et al., 2010), the subscales have been characterized by much weaker internal consistency scores (e.g., Lambert et al., 2002; Lo Coco et al., 2008). These cross-country variations, combined with the heterogeneity in the number of factors, lead us to investigate the behavior of the measure in the Hungarian context. With the results obtained, we would like to further nuance the academic discourse on the cultural and geographical determinants of the questionnaire. In addition to examining general psychometric indicators, we also aim to gain insight into the factor structure of the instrument during validation.

From the outset, the evaluation of the OQ-45 questionnaire has been heavily influenced by the comparison of the scale with the depression construct, with the *Symptom Checklist-90-Revised* (Lambert et al., 1996) and the *Depressive, Anxious, and Somatoform Disorders Measuring Scale* (Alvarado et al., 1991) confirming the hypothesized convergence (*r* = 0.72; *r* = 0.83;). In addition, questionnaires measuring interpersonal relationships have been used extensively for the external validation of the OQ-45 scale, including the *Inventory and Interpersonal Problem (IIP)* and *The Social Adjustment Rating Scale (SAS)* (e.g., Lambert et al., 2002; de Jong et al., 2007; Wennberg et al., 2010) The correlations with the full scale are smaller than for depression (r_IIP_ = 0.59–0.63; r_SAS_ = 0.58–0.67) but strong enough to consider these questionnaires as convergent with the OQ-45 scale.

Our hypothesis for convergent validity, based on the literature, is that the *Outcome Questionnaire-45* (OQ-45) will show (1) positive correlations with *depression,* (2) negative correlations with *resilience*, (3) negative correlations with *well-being,* and (4) negative correlations with *social support.*

## 2 Method

### 2.1 Procedure and sample presentation

The data for validation were provided by the 2021 survey organized by the Hungarian Association for Counseling in Higher Education (FETA), which aimed to investigate the mental well-being of active-status university students in Hungary through a questionnaire. The translation into the Hungarian language was carried out by two independent, bilingual translators using back-translation (Brislin, 1970). The investigation was approved by the Research Ethics Committee of the Faculty of Education and Psychology of Eötvös Loránd University (ethic approval number: 2021/357).

The data collection started in October 2021, when the questionnaire package was sent by e-mail to the rectors and chancellors of all Hungarian higher education institutions, the staff of the institutional higher education student advisors, the members of FETA and the HÖOK (the democratic representation of the student self-governments of Hungarian higher education institutions). The university managers and staff of the organizations contacted were asked to post the link and the invitation to the online questionnaire on their institutional mailing lists and electronic study systems, or on any other channel they use. At the same time, students were encouraged to complete the questionnaire on the FETA website and social media pages (Facebook). Before completing the online questionnaire package, subjects declare that they have read the information statement, understand its contents (they are participating in the research voluntarily, they can stop completing the questionnaire at any time without giving any reason), and agree to the use of the collected data in an anonymous manner. Several institutions requested an extension of the data collection period, which ended on December 6 instead of the planned November 30, 2021.

More than 10,000 students (10,196) from 47 Hungarian higher education institutions completed the online questionnaire. The response rate was over 75%, and data analysis was conducted exclusively on the complete responses received (*N* = 7,695), without any attempt to fill in missing data. The gender distribution of the sample was as follows: 68% female; 28.6% male; 1% other; 2.4% did not wish to specify their gender identity. According to the latest data from the Hungarian Central Statistical Office, the proportion of women in higher education (54%) is only a few percentage points higher than that of men (46%), but in our sample the difference seems to be much more pronounced in favor of women. The gender distribution of the respondents, like other variables such as educational levels (e.g., Bachelor’s, Master’s, or Ph.D.) does not aim to represent the entirety of the Hungarian higher education population. The mean age of the respondents was 23, 7 years (SD = 6.78). The vast majority of students (96.8%) had a single major or program, 3% of the sample had two, and 0.2% had more than two majors/programs. Of the students, 84.8% are full-time students and 13.9% are part-time and distance learners (together representing just over 1% of the sample). Inclusion criteria for the sample were active student status and age over 18. Although the university sample can be considered heterogeneous along demographic, gender, and college variables, our study does not include the clinical population, which is a limitation of our research.

### 2.2 Measuring instruments

The most important part of the questionnaire package was the *Outcome Questionnaire-45 (OQ-45)* to be validated, which was also licensed to us by the test developer (Lambert). In selecting the additional questionnaires to be included in our research, we aimed to use scales that were suitable for testing convergent validity and for conducting regression analyses, i.e., scales that measure constructs related to the *OQ-45*. Another consideration was that these questionnaires are widely used abroad, are reliable and have been adapted into Hungarian. Their advantages lie in their brevity, comprehensibility and ease of scoring. All these factors facilitated both the preparation and the data collection phases of the research.

*Outcome Questionnaire-45 (OQ-45)* (Lambert et al., 1996), originally a 3-factor instrument measuring symptom distress, interpersonal relationships and social roles, was described in detail in the theoretical introduction.

- In the *demographic data* block, questions about gender, age, place of residence, institution of higher education, and educational background were formulated for the descriptive statistical analysis of our sample.
- *The Beck Depression Inventory (BDI)* (Beck, 1972) is a widely used instrument for measuring the level of mood, especially the severity of depressive symptoms. The 9-item, 4-point (Likert scale) version of the scale (Kopp et al., 1990), adapted to the Hungarian language, has reliable standards in terms of psychometric indicators (Rózsa et al., 2001).
- *Resilience* is a flexible adaptability of one’s personality that facilitates successful adaptation to difficult life circumstances, enables coping with adversity, and mitigates the negative effects of stress (Connor and Davidson, 2003). In our research, we used the 10-item shortened Hungarian version (Járai et al., 2015) of the 25-item *Connor-Davidson Resilience Scale (CDRISC)* (2003) to measure the student sample It was completed by rating the frequency of statements on a 5-point Likert scale (1: not at all true, 5: almost always true) for the past month.
- The *WHO Wellbeing Questionnaire* (Bech et al., 1996) is a widely used measure of psychological well-being (e.g., cheerfulness and happiness) experienced by the respondent in the past two weeks. The questions are answered on a 4-point Likert scale. A shortened version of the Hungarian adaptation (*WBI-5*) of the 5 statements was developed in a national population health survey (Susánszky et al., 2006) and we use this version in the present study.
- *Social support* is the ability of an individual to rely on his or her peer environment in times of difficulty. Empirical evidence shows a strong association between perceived social support and physical and psychosocial health and psychological well-being (Caldwell et al., 1987; Giangrasso and Casale, 2014). The *MOS SSS-H – Medical Outcomes Study* (Sherbourne and Stewart, 1991) *Social Support Survey* was chosen to investigate this construct. It can be used to assess the extent to which a participant feels that there is a person they can rely on in difficult life situations (e.g., illness, depression). The instructions for completion asked participants to indicate how much they could rely on different forms of support on a 5-scale (1-not at all, 5-totally). The Hungarian version was adapted by Szentiványi-Makó et al. (2016), but it was less adaptable to our sample of university students, so we reverted to the original version in the present study. To translate it into English, here too, we used the back-translation method (Brislin, 1970).
- The phenomenon of *burnout* was first observed in health care workers (Freudenberger, 1974) and has since been studied in a variety of populations. Since burnout in students can contribute to a deterioration in academic performance and can also lead to psychological problems (anxiety, depression), it is considered content relevant to several constructs measured by the *OQ-45* instrument. Its conceptualization for students is attributed to Schaufeli et al. (2002), and the Hungarian adapted version (Hazag et al., 2010) of his questionnaire (*Maslach Burnout Inventory-Student Version; MBI-SS*) has reliable and valid standards, so we chose this instrument for convergent validation in the context of burnout. The 15 items are based on three factors: 1. *Exhaustion* (physical, emotional, and mental due to the demands of studying); 2. *Cynicism* (and the detachment from one’s studies and the profession); 3. *Loss of performance and efficiency.*

### 2.3 Data analysis

Data were processed using IBM SPSS Statistics (RRID:SCR_016479) and the JASP (RRID: SCR_015823) statistical software package. The internal consistency of the instrument was interpreted using the Cronbach’s alpha coefficient (correlation between mean scores). Confirmatory factor analysis, principal component analysis and exploratory factor analysis were performed on our entire sample to find the factor structure that best fit the sample. To describe the models, we used fit indicators (e.g.: CMIN/df; CFI; TLI; RMSEA) commonly accepted in the literature. In addition to using the full scale of the *OQ45* questionnaire package for convergent and divergent validation, subscale and full scale scores were calculated and included in the analysis, as well as scale and total scores from the *Beck Depression Inventory (BDI), Connor-Davidson Resilience Scale (CDRISC), WHO-Well-being Inventory-5 (WHO-WBI-5), Social Support Survey* (*MOS-SSS-H)*. Pearson correlation and multiple linear regression analyses were used to compare the scales and the different factors.

## 3 Results

### 3.1 Features of the OQ-45

Table 1 shows the respondents’ mean scores on the OQ total scale and subscales, as well as the internal consistency of each scale. The Cronbach’s alpha (α_total_) for the 45-item full scale in our sample is 0.95. Along the same indicator, the SD subscale is 0.94, the IR subscale is 0.76 and the SR subscale is 0.67. These results show a similar pattern to the original questionnaire developed by Lambert (α_SD_ = 0.91; α_IR_ = 0.74; α_SR_ = 0.71).

**Table 1:** Descriptive statistics and internal consistency of the OQ scale.

Scale	Mean	SD	Cronbach’s-alfa	Items that do not fit on the scale because of the low CITC index
OQ Symptom Distress (SD)	73,038	29,089	0,94	OQ-11
OQ Interpersonal Relations (IR)	15,4	7,64	0,76	OQ-19
OQ Social Role (SR)	11,5	4,43	0,67	OQ-14, OQ-32
OQ Total score	73,04	29,089	0,95	OQ-11, OQ-14, OQ-19, OQ-32

In terms of item behavior, the fit of 4 items to the scale is not considered adequate due to low CITC values (<0.3) (OQ11_CITC_ = 0.156; OQ14_CITC_ = 0.062; OQ19_CITC_ = 0.288; OQ32_CITC_ = 0.153; (Table 1). Poor fits for OQ11 (*After heavy drinking, I need a drink the next morning to get going*) and OQ32 (*I have trouble at work/school because of drinking or drug use*) have also been described in the international literature (de Jong et al., 2007; Lo Coco et al., 2008). Despite the statistical arguments, the authors consider it justified to retain both items because of their indicative function (alcohol and substance abuse). For the same reason, we also included these items in our final questionnaire (referred to as critical items), but separated them from the subscales. Item OQ14 (*I work/study too much*) is also considered a culturally embedded item in the international literature (e.g., de Jong et al., 2007), but in our study the low item-total correlation may be explained by the specific student sample. In the case of the OQ19 (*I have frequent arguments*) there may also be a biasing effect of the university sample. It is conceivable that for this population, “arguing” does not necessarily imply conflict or a tense relationship, so that when interpreting the statement, respondents may think of frequent clashes of arguments or intellectual discourse.

### 3.2 Factor structure of the questionnaire

#### 3.2.1 Confirmatory factor analysis (CFA)

The international literature reports very heterogeneous results in terms of factor structure (different subscales ranging from 1 to 10 were found). In order to understand the factor structure of our sample, we first performed a confirmatory factor analysis to examine the structure of the original questionnaire (three-factor).

To check the prerequisites, we tested the significance level of the correlation matrix and calculated a KMO value, all of which were found to be sufficient for further analysis (KMO = 0.969; *p* < 0. 001). However, as shown in Table 2 more details, since none of the goodness-of-fit indicators were found to be sufficient (CFI = 0.773, TLI = 0.761, RMSEA = 0.073) we could not confirm the applicability of the three-factor model to our sample. Another statistical argument for rejecting the three-factor models is the high correlation between the factors (*r* = 0.69–0.91), which may represent an insufficient separation of the subscales from each other.

**Table 2:** Indicators of the CFA fit the sample.

Model	χ^2^	df	χ^2^/df	CFI	TLI	RMSEA	SRMR
3 factor solution	169654.48	990	171.36	0.773	0.761	0.073	0.061

The results obtained are in line with the experience of international studies, which show that the original structure could be confirmed with no or only moderate goodness of fit (e.g., Mueller et al., 1998; de Jong et al., 2007). The results of the CFA analyses in the same study warranted further exploratory analyses to identify alternative models that better fit the sample. We conducted our data analysis along these lines using exploratory factor analysis (EFA).

#### 3.2.2 Principal component analysis (PCA) and exploratory factor analysis (EFA)

To determine the number of factors, a Principal Component Analysis (PCA) was first performed, which revealed 8 factors with eigenvalues greater than 1. Since the factor loadings indicated that the factors contained items with different item numbers, the internal consistency of the factors was first examined. Cronbach’s alpha reached the required value (>0.75) for the first 5 factors to be considered reliable (F1_α_ = 0.9; F2_α_ = 0.92; F3_α_ = 0.87; F4_α_ = 0.73, F5_α_ = 0.75) and the fit of the items was found to be sufficient in all cases (CITC >0.4). Due to the low consistency of the additional factors (F6_α_ = 0.63; F7_α_ = 0.19, F8_α_ = 0.4), an attempt was also made to explore an alternative factor structure (EFA). Items that did not fit into any of the factors (OQ6, OQ16) were not included in this analysis. Due to low Cronbach’s alpha values (< 0.75) further items were also dropped (OQ4, OQ11, OQ14, OQ26, OQ32). On the 38 items remaining in the analysis, 6 components were identified by PCA and EFA analysis. The reliability of the first 5 factors still exceeded the required threshold (>0.75), but the internal consistency of factor 6 was found to be weak (α = 0.56) and the items loading here (OQ19, OQ39, OQ44) were also dropped.

Thus, the item reduction process reduced the original 45-item instrument was narrowed down to 35 items, all of which fit perfectly into the 5 factors identified in the EFA (Table 3) [Of the dropped items, four items (OQ11, OQ26, OQ32, OQ44) were later reinserted into the final item list after data analysis because of their signaling function, and these items are not included in further data analysis, but are an important subset of the instrument].

**Table 3:** Factor loadings in a five-factor model; after deletion of non-matching items.

Item No. OQ-45	Item No. OQ-39	Item	Factor 1	Factor 2	Factor 3	Factor 4	Factor 5
OQ-10	8	I feel fearful	0.667				
OQ-15	12	I feel worthless	0.661				
OQ-5	4	I blame myself for things	0.653				
OQ-33	28	I feel that something bad is going to happen	0.623				
OQ-25	20	Disturbing thoughts come into my mind that I cannot get rid of	0.613				
OQ-40	34	I feel something is wrong with my mind	0.592				
OQ-8	6	I have thoughts of ending my life*	0.575				
OQ-23	18	I feel hopeless about the future	0.548		0.443		
OQ-9	7	I feel weak	0.54		0.430		
OQ-24	19	I like myself	0.521	0.489			
OQ-1	1	I get along well with others		0.73			
OQ-43	37	I am satisfied with my relationships with others		0.729			
OQ-20	15	I feel loved and wanted		0.661			
OQ-37	32	I feel my love relationships are full and complete		0.614			
OQ-30	25	I have trouble getting along with friends and close acquaintances		0.61			
OQ-31	26	I am satisfied with my life	0.418	0.591			
OQ-13	11	I am a happy person		0.588			
OQ-21	16	I enjoy my spare time		0.486			
OQ-22	17	I have difficulty concentrating			0.711		
OQ-28	23	I am not working/studying as well as I used to			0.692		
OQ-38	33	I feel that I am not doing well at work/school			0.66		
OQ-3	3	I feel no interest in things			0.632		
OQ-12	10	I find my work/school satisfying		0.417	0.567		
OQ-2	2	I tire quickly			0.546	0.437	
OQ-42	36	I feel blue			0.498	0.416	
OQ-34	29	I have sore muscles				0.678	
OQ-45	39	I have headaches				0.644	
OQ-27	22	I have an upset stomach				0.642	
OQ-29	24	My heart pounds too much				0.618	
OQ-35	30	I feel afraid of open spaces. of driving. or being on buses. Subways. and so forth				0.471	
OQ-36	31	I feel nervous	0.417			0.462	
OQ-41	35	I have trouble falling asleep or staying asleep				0.452	
OQ-17	13	I have an unfulfilling sex life					0.799
OQ-7	5	I feel unhappy in my marriage/significant relationship					0.659
OQ-18	14	I feel lonely					0.623

### 3.3 Cross-validation

The instrument was also cross-validated, allowing exploratory and confirmatory factor analysis to be conducted on two subsets of the same sample. We believed that this would provide further support for the questionnaire design described above by using a method that is considered reliable in the literature (de Rooij and Weeda, 2020). The sample was randomly divided into two groups, the first sub-sample was subjected to PCA and EFA and the second sub-sample was subjected to CFA analysis. Based on the principal component analysis, the eigenvalues of 5 factors took values higher than 1. Since the last two factors (4 to 5) already contributed very little variance to the model (< 3.2%), the CFA analysis was performed for a 3-factor solution in addition to the 5-factor model, which is summarized in Table 4. Again, the final model was adopted based on the values of the goodness of fit indices that were found to be the most appropriate for the 5-factor structure (CFI = 0.887; TLI = 0.877, RMSEA = 0.062).

**Table 4:** Indicators of the CFA fit on the subsample.

Model	χ^2^	df	χ^2^/df	CFI	TLI	RMSEA	SRMR
3 factor solution	72919.3	595	122.55	0.859	0.849	0.069	0.053
5 factor solution	68315.68	561	121.77	0.887	0.877	0.062	0.0050

### 3.4 Content analysis

The statistical analysis was followed by a content analysis of the items. Based on their meanings, the fit of the 8 items that loaded highly on multiple factors to subscales was examined one by one, and a decision was made on their final ranking.

The OQ23 (*I feel hopeless about the future*) loaded on Factors 1 and 3 at almost the same rate (0.54, 0.44) that we assigned to list of items that included anxiety, as we felt it was closer in content. OQ9 (*I feel weak*) also loaded highly on Factor 1 and Factor 3 (0.54, 0.43), but in the content analysis we placed this item on Factor 1. The reason for our decision was that Factor 3 consists of very homogeneous items (work, performance) from which seemed better to separate this item. For OQ24 (*I like myself*) we had to choose between Factor 1 and Factor 2 (0.52, 0.48). We believe that the relationship with the inner world is better captured by Factor 1, so we placed this item here. Of Factors 1 and 2, we chose the latter (0.48, 0.59) for OQ31 (*I am satisfied with my life*) because the meaning of this scale better captures satisfaction, the cognitive evaluation of feelings. For OQ12, there was a cross-loading on factors 2 and 3 (0.41, 0.56). Factor 3 has already been mentioned above as a homogeneous scale, so we clearly place the item OQ12 (*I am satisfied with my work/school*) related to school performance on this factor. On the other hand, the item OQ2 (*I tire quickly*) (0.54, 0.43) and OQ42 (*I feel blue*) (0.49, 0.41), which load on factors 3 and 4, and OQ36 (*I feel nervous*) (0.42, 0.46), which load on factors 1 and 4, were also placed on subscale 4 (physical symptoms).

To compare the fit of our 5-factor model before and after the content reclassification, we again used the confirmatory factor analysis (CFA) method. Although in both cases the CFI values (CFI > 0.8; TLI > 0.85; RMSEA<0.08) were considered satisfactory, the better fit of the model after content analysis (CFI = 0.89; TLI = 0.88; RMSEA = 0. 062) also provides methodological justification for the placement of cross-loading items on our factors compared to the pre-reclassification structure (CFI = 0.88; TLI = 0.88; RMSEA = 0.064).

Thus, after statistical and content analysis, 35 items from the original 45 item questionnaire were placed into 5 subscales, which were given the following titles based on the meaning of the items to which they corresponded: *1. Anxiety, Relationship with the inner world, Hopelessness (ARH); 2. Congruence (authenticity, consistency of self-image and experiences) and Relationship with others (CR); 3. Lack of performance, Work, Interest, Burnout (LWIB); 4. Physical discomfort and Symptomatology (PS); 5. Intimacy (I)*. The subscales were supplemented by four critical items that did not fit the factors (OQ32, OQ11, OQ26 for substance abuse; OQ44 for aggression). Thus, our instrument, adapted to the final Hungarian model, thus consists of 39 items and is called OQ-39. Table 5 shows the items and scales of the original questionnaire and the Hungarian version, illustrating their differences and similarities, as well as the process of item reduction and content analysis.

**Table 5:** Original and final item list after statistical and content analysis.

Item	OQ-39 item No.	OQ-39 subscale	OQ-45 item No.	OQ-45 subscale
I get along well with others	1	CR	OQ-1	IR
I tire quickly	2	PS	OQ-2	SD
I feel no interest in things	3	LWIB	OQ-3	SD
I blame myself for things	4	ARH	OQ-5	SD
I feel unhappy in my marriage/significant relationship	5	I	OQ-7	IR
I have thoughts of ending my life*	6	ARH	OQ-8	SD
I feel weak	7	ARH	OQ-9	SD
I feel fearful	8	ARH	OQ-10	SD
After heavy drinking. I need a drink the next morning to get going. (If you do not drink. Mark “never”)*	9	-	OQ-11	SD
10. I find my work/school satisfying	10	LWIB	OQ-12	SR
11. I am a happy person	11	CR	OQ-13	SD
I feel worthless	12	ARH	OQ-15	SD
I have an unfulfilling sex life	13	I	OQ-17	IR
I feel lonely	14	I	OQ-18	IR
I feel loved and wanted	15	CR	OQ-20	IR
I enjoy my spare time	16	CR	OQ-21	SR
I have difficulty concentrating	17	LWIB	OQ-22	SD
I feel hopeless about the future	18	ARH	OQ- 23	SD
I like myself	19	ARH	OQ- 24	SD
Disturbing thoughts come into my mind that I cannot get rid of	20	ARH	OQ-25	SD
I feel annoyed by people who criticize my drinking (or drug use) (If not applicable. Mark “never”)*	21	-	OQ-26	IR
I have an upset stomach	22	PS	OQ-27	SD
I am not working/studying as well as I used to	23	LWIB	OQ-28	SR
My heart pounds too much	24	PS	OQ-29	SD
I have trouble getting along with friends and close acquaintances	25	CR	OQ-30	IR
I am satisfied with my life	26	CR	OQ-31	SD
I have trouble at work/school because of drinking or drug use*	27	-	OQ-32	SR
I feel that something bad is going to happen	28	ARH	OQ-33	SD
I have sore muscles	29	PS	OQ-34	SD
I feel afraid of open spaces. of driving. or being on buses. Subways. and so forth.	30	PS	OQ-35	SD
I feel nervous	31	PS	OQ-36	SD
I feel my love relationships are full and complete	32	CR	OQ-37	IR
I feel that I am not doing well at work/school	33	LWIB	OQ-38	SR
I feel something is wrong with my mind	34	ARH	OQ-40	SD
I have trouble falling asleep or staying asleep	35	PS	OQ-41	SD
I feel blue	36	PS	OQ-42	SD
I am satisfied with my relationships with others	37	CR	OQ-43	IR
I feel angry enough at work/school to do something I might regret*	38	-	OQ-44	SR
I have headaches	39	PS	OQ-45	SD
Deleted items				
I feel stress at work/school	–	–	OQ-4	SR
I feel irritated	–	–	OQ-6	SD
I work/study too much	–	–	OQ-14	SR
I am concerned about family troubles	–	–	OQ-16	IR
I have frequent arguments	–	–	OQ-19	IR
I have too many disagreements at work/school	–	–	OQ-39	SR

The stars indicate critical items that have a signaling function.

Although the correlation between the factors remains high even with the omission of the items, the degree of cointegration shows a significant decrease (0.3–0.7). Beyond the statistical explanations, the factors modified and finalized in the content analyses form a well-defined group in their meaning. In terms of international results, this structure is closest to the Dutch sample study, as De Jong et al. (2007) also identified five factors. Similar to our findings, their questionnaire included anxiety and its physical, somatic symptoms, which were also included on a scale in the original instrument, as separate factors. An important difference, however, is that the *Social Roles (SR)* scale consists of only one item in the Dutch adaptation. This raises the question of the justification of separating or even keeping the whole factor. In our study, there is a greater balance in the number of items that make up the factors, with the *Intimacy (I)* subscale, which has the fewest items, also having three items.

### 3.5 Correlation of the questionnaire with other measures

#### 3.5.1 Convergent validity

Convergent validity aimed to compare the results of the OQ-39 we developed with those of other widely used measures of similar constructs on the same sample. Our preliminary hypotheses were that the constructs of *depression, resilience, well-being, and social support* would be convergently related to the currently tested OQ-39 scale, and accordingly we used data from the *Beck Depression Inventory (BDI), Connor-Davidson Resilience Scale (CDRISC), WHO-Well-being Questionnaire (WHO-WBI-5), Social Support Survey* (*MOS-SSS-H*) questionnaires to test our hypotheses. (The correlations between the questionnaires used for convergent validity testing and the OQ-39 scale and its subscales are summarized in Table 6).

**Table 6:** Current validity estimates for the OQ-39 with BDI, DCRISC, WHO-WBI-5, MOS-SSS-H, and MBI-SS.

	BDI	DCRISC	WHO-WBI-5	MOS-SSS-H	MBI- SS
1. Anxiety, Relationship with the inner world, Hopelessness (ARH)	0.834	−0.622	−0.647	−0.39	0.63
2. Congruence and Relationship with others (CR)	0.703	−0.591	−0.68	−0.63	0.54
3. Lack of performance, Work, Interest, Burnout (LWIB)	0.73	−0.608	−0.637	−0.3	0.76
4. Physical discomfort and Symptomatology (PS)	0.707	−0.418	−0.591	−0.26	0.54
5. Intimacy (I)	0.487	−0.337	−0.439	−0.53	0.32
OQ-39 Total score	0.863	−0.637	−0.73	−0.493	0.684

All correlation is significant; BDI, Beck Depression Inventory; CDRISC, Connor-Davidson Resilience Scale; WHO-WBI-5, WHO-Well-being Questionnaire; MOS-SSS-H, Medical Outcome Study-Social Support Survey; MBI-SS, Maslach Burnout Inventory-Student Version.

The *BDI* total score, which indicates the degree of depression, showed a rather high significant correlation (>0.49) with each of the OQ-39 factors, with the strongest correlation being with Factor *1, Anxiety, Relationship to the inner world, Hopelessness (ARH)* (*r* = 0.834) and the OQ-39 total score (*r* = 0.863). A very similar relationship can be observed for the *Resilience (CDRISC)* total score. This construct was also significantly correlated with the OQ-39 total scale (*r* = −0. 64) and all its factors (although in the opposite direction to the previous depression variable), with the strongest correlation being with factor 1 (*r* = −0. 62). The *WHO (WBI-5) well-being* score was also significantly – and also negatively – correlated with both the OQ-39 scale (−0.0.73) and its subscales, with the strongest correlation (−0.68) for the *Congruence and Relationship with others (CR) subscale.*

The *MOS-SSS-H* score (measure of social support) showed a significant inverse relationship with each factor of the OQ39, with the strongest associations being with Factor 2 (*CR*) and Factor 5 (*I*) (r_CR_ = −0.63, r_I_ = −0.53, respectively).

According to the factor analysis and content analysis, the most homogeneous factor of the OQ39 scale is the third factor, each item of which captures a dominant aspect of work/school performance and motivation. For this reason, although we did not formulate a specific hypothesis, we considered it important to see how this subscale related to the *Maslach Burnout Inventory-Student Version (MBI-SS)* questionnaire, which was specifically designed and standardized to measure burnout among higher education students. Correlation analyses showed that the *MBI-SS* total score was correlated with all the factors of the OQ39, most strongly with Factor 3 (*LWIB*) (*r* = 0.76). In addition to the content argument, the obtained result can be considered as a methodological reference for the validation of the burnout subscale.

#### 3.5.2 Regression

In the linear regression analysis, we wanted to see whether any of the variables (depression, resilience, well-being, social support) formulated in our hypothesis and already tested in the convergent validity test could predict OQ-39 scores beyond their interaction with the OQ scale. The close relationship between burnout and the OQ-39 scale revealed in the present work justified our inclusion of this construct in the regression model. The analysis was performed using the stepwise method, with the items included in the model in order of increasing explanatory power: *Beck Depression Inventory (BDI)* total score, *WHO-Well-Being Questionnaire (WHO-WBI-5)* total score, *Social Support (MOS-SSS-H*) total score, *Maslach Burnout Inventory-Student Version (MBI-SS)* total score, *Resilience Scale (CDRISC)* total score.

All 5 models are considered significant (*p* < 0.05) and the Durbin-Watson pretest value (1.97) is within the acceptable range (1.5–2.5). Model 1 is 75% (*BDI*_total_, R = 0.863), followed by Model 2 (*BDI*_total_, *WHO-WBI-5*_total_, R = 0.891) with 79.4%, Model 3 (*BDI*_total_, WHO-WBI-5 _total_, *MOSS-SSS-H*_total_; R = 0.897) with 80.5%, Model 4 (*BDI*_total_, *WHO-WBI-5*_total_, *MOSS-SSS-H*_total_ and *MBI-SS*_total_, R = 0.905) with 81.8%, Model 5 (*BDI*_total_, WHO _total_, *MOSS-SSS-H*_total_ and *MBI-SS*_total_, *CDRISC*_total_, R = 0.906) explains 82.2% of the dependent variable (OQ-39 total).

## 4 Discussion

The aim of the present study was to psychometrically evaluate and validate the *Outcome Questionnaire-45* (OQ-45; Lambert et al., 1996) in a Hungarian sample. The relevance of the study was due, among other things, to the large sample size and the practical application of previous versions of the questionnaire (mainly for measuring therapeutic effects). However, it was felt that the advantages of the instrument (low cost, easy to administer) also allowed it to have a number of untapped potentials in the field of psychological research (e.g., cross-sectional state assessment). Previous studies have also drawn attention to the cultural determinants of the instrument, which provided us with an additional argument for exploring the behavior and factor structure of the instrument in an Eastern European context.

Based on the reliability analysis, the questionnaire was found to be reliable, with high internal consistency, similar to the original US and foreign adaptations. Confirmatory factor analysis failed to confirm the original 3-factor structure of the questionnaire in a similar way to international studies. Further exploratory analyses identified 5 distinct factors, and the results were supported by cross-validation. Items that did not fit into subscales were dropped, and the final placement of items that loaded equally on multiple factors was decided on the basis of content analysis. The factors were named after the corresponding items: *1. Anxiety, Relationship with the inner world, Hopelessness (ARH), Hopelessness; 2. Congruence (authenticity, consistency of self-image and experiences) and Relationship with others (CR); 3. Lack of performance, Work, Interest, Burnout (LWIB); 4. Physical discomfort and Symptomatology (PS); 5. Intimacy (I).*

The final questionnaire consists of 39 items, 4 of which do not fit into any of the above subscales, but are a dominant part of the instrument because of their signaling function (Table 5).

A convergent validity test was also conducted to further define the psychometric properties of the questionnaire. Our hypotheses regarding the correlations were confirmed by the correlation and regression results, the shortened version of the *OQ-45 Questionnaire (OQ-39)* developed by us and adapted to the Hungarian sample is positively related to depression and burnout, and negatively related to the variables of social support, resilience and well-being. These associations are consistent with international research findings in several respects. The *Beck Depression Inventory (BDI),* most commonly used in *OQ-45* validation studies with the *State–Trait Anxiety Inventory (STAI*) and the *Symptom Checklist-90 -Revised (SCL) OQ-45* total score, showed convergence (0.71–0.84) when measured in different populations (e.g., Lambert et al., 1996; Umphress et al., 1997). The more nuanced correlations we found are also similar to those found in the literature, which found that Factor 1 (SD) of the original questionnaire correlated most strongly (0.65–0.84) with convergent measures (Chapman, 2003). In our study, the strongest relationship was also found between the *BDI* and the *Anxiety, Relationship to the inner world, Hopelessness (ARH)* factor. The regression model also confirms this finding, showing that in our data, depression seems to be strongest predictive variable in the OQ-39 scale. Overall, therefore, the full scale and subscales of the OQ-39 were highly correlated with similar constructs.

Perhaps the greatest strength of the study is its comprehensive description of psychometric characteristics. The results obtained are further strengthened by the large sample size, making them even more reliable. The results of the validation of the questionnaire may also be relevant in an Eastern European context. In this region, only one Polish-language adaptation (Simon et al., 2015) has been produced so far, which differs from our work both in terms of sample characteristics (number of items, population surveyed) and research focus. The construct validity of the questionnaire may make it unnecessary to include several convergent measures at the same time, thus eliminating many of the methodological limitations that arise when constructing larger questionnaire packages. As the OQ-39 appears to be able to provide a comprehensive cross-sectional picture of the bio-psycho-social status of individuals in a population that can be considered highly heterogeneous (university students), it is also suitable for systematic monitoring of mental health indicators. The emergence of student counseling centers in Hungarian higher education institutions has been on the rise in recent years, with some form of mental health service now available in most places. However, due to limited financial and human resources, there are often long waiting lists, meaning that students have to wait weeks or even months to access services. Capacity constraints also mean that the number of sessions is almost always limited. We believe that the use of this tool, which can be used to assess condition and measure change, can be a great help to professionals in working effectively when deciding on interventions.

In addition to being one of the largest populations at the national level, university students are also the future workforce, and the results of research on them can be very informative and useful from a public health, economic and policy perspective. It is hoped that the findings of these studies will not only raise awareness of the importance of studying the university population, but may even provide an argument for expanding (and resourcing) university mental health services at both the prevention and intervention levels.

Of course, if conclusions are to be drawn for the Hungarian population as a whole, it may be worthwhile in the future to extend the study to a more heterogeneous sample covering a wider age range. There are also further possibilities to compare clinical and subclinical groups.

## Data availability statement

The original contributions presented in the study are included in the article/supplementary material, further inquiries can be directed to the corresponding author.

## Ethics statement

The studies involving humans were approved by Research Ethics Committee, Faculty of Education and Psychology, ELTE Eötvös Loránd University, Budapest, Hungary. The studies were conducted in accordance with the local legislation and institutional requirements. The participants provided their written informed consent to participate in this study.

## Author contributions

DM: Conceptualization, Formal analysis, Methodology, Project administration, Validation, Writing – original draft. LN: Conceptualization, Methodology, Supervision, Writing – review & editing. OK: Conceptualization, Investigation, Methodology, Supervision, Writing – review & editing.
